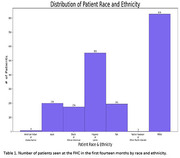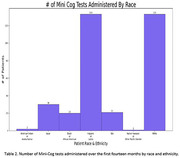# Electronic Health Record‐Embedded Dementia Screening Toolkit Tailored for Bilingual and Bicultural Needs

**DOI:** 10.1002/alz.093016

**Published:** 2025-01-09

**Authors:** Samantha Shah, Gabriela Islas Huerta, Kevin Wang, Satpal S. Wadhwa, Mirella Diaz‐Santos, Keith Vossel, Timothy S Chang

**Affiliations:** ^1^ David Geffen School of Medicine, University of California, Los Angeles, Los Angeles, CA USA; ^2^ David Geffen School of Medicine at UCLA, Los Angeles, CA USA; ^3^ University of California, Los Angeles, CA USA; ^4^ Movement Disorders Programs, Department of Neurology, David Geffen School of Medicine, University of California, Los Angeles, Los Angeles, CA USA

## Abstract

**Background:**

Alzheimer’s disease (AD) is a significant health concern affecting at least 10% of individuals aged 65 and older, with heightened risk in Black and Hispanic/Latino populations. Despite this prevalence, our analysis of University of California Los Angeles (UCLA) electronic health records (EHR) indicates that only 4% of patients aged 65 or older receive an AD diagnosis, with underdiagnosis more prevalent among Black and Hispanic/Latino patients compared to their white counterparts. To address this issue, we propose implementing a concise dementia screening tool (DST) in real‐world clinical settings.

**Method:**

The DST, developed through collaborative efforts among multiple University of California AD Centers and the California Department of Public Health, is a brief (<5 minutes) screening tool. It includes a three‐question questionnaire for patients and, if available, their informants, followed by the Mini‐Cog assessment if applicable. The questions address changes in language, memory, and personality. A positive DST result is defined as any “Yes” response by the patient or informant to any question or a Mini‐Cog score below 3 out of 5 points. We have also created a Spanish version of the questionnaire tailored to the Hispanic/Latino cultural context.

**Result:**

Integration of the DST into the EHR system facilitates a seamless implementation. Patients receive questionnaire through the EHR messaging system before appointments, with the Spanish version available for those who prefer it. Responses and Mini‐Cog results are easily accessible in the EHR. Additionally, a documentation link automatically populates DST results, final scores, and recommendations into provider notes.

The DST is currently in use at a large, diverse UCLA family medicine clinic serving Hispanic/Latino and Black patients. In the first fourteen months, 1,963 patients (Table 1) were seen, with 28% self‐identifying as Hispanic or Latino. Among them, 1,070 patients answered at least one question, and 340 completed the Mini‐Cog (Table 2). Notably, 31 patients received a new dementia diagnosis, and 81 were referred to Neurology.

**Conclusion:**

Our implementation of the DST in a diverse clinical setting has successfully screened 1,070 participants, with future research comparing data from the DST period with a pre‐time period.